# Rethinking the evidence on COVID-19 in Africa

**DOI:** 10.1016/S1473-3099(25)00071-4

**Published:** 2025-04-04

**Authors:** Philip Bejon, Ambrose Agweyu, L Isabella Ochola-Oyier, Mainga Hamaluba, Dorcas Kamuya, Sam Kinyanjui, Edwine Barasa

**Affiliations:** KEMRI-Wellcome Trust Research Programme, Centre for Geographical Medicine Research (Coast), Kilifi, Kenya; Modernising Medical Microbiology Nuffield Dept of Medicine, University of Oxford, Oxford, UK; KEMRI-Wellcome Trust Research Programme, Centre for Geographical Medicine Research (Coast), Kilifi, Kenya; Department of Infectious Disease Epidemiology and International Health, London School of Hygiene and Tropical Medicine, London, UK; KEMRI-Wellcome Trust Research Programme, Centre for Geographical Medicine Research (Coast), Kilifi, Kenya; Centre for Tropical Medicine and Global Health Nuffield Dept of Medicine, University of Oxford, Oxford, UK

## Abstract

The COVID-19 pandemic was predicted to cause substantial mortality in Africa. However, some countries in Africa had a striking absence of overwhelmed hospitals and low reported mortality. The marked contrast with the overwhelmed hospitals and high mortality seen in Europe and other high-income settings was regarded as puzzling and a paradox. In this Review, we reflect on possible explanations for the paradox with particular reference to observations made on the ground in Kenya. The evidence is inconsistent with reduced viral transmission or poor surveillance as primary explanations for the discrepancy. Population age structure is an important but incomplete explanation of the epidemiology. Due to the high prevalence of asymptomatic infection, low mortality, and evidence of reduced inflammatory responses, we hypothesise that some populations in Africa might have reduced susceptibility to symptomatic COVID-19. The reduced inflammatory responses might result from immunoregulation or cross-reactive, pre-pandemic cellular immunity, although the evidence is not definitive. Local data are essential to develop public health policies that align with the reality on the ground rather than external perceptions.

## Introduction

The first cases of COVID-19 in Africa were reported in Egypt and Nigeria in Feb, 2020, and in Kenya in March, 2020. COVID-19 cases were subsequently identified in all parts of Africa. International agencies were guided by analyses predicting millions of COVID-19-related deaths in Africa^1^ and advised that few options were available for mitigation of the impending disaster on the continent.^2^ Many countries in Africa responded by taking immediate firm action, including lockdowns and social restrictions, and considered the attendant economic and public health costs to be justified by the impending disaster.^3^ In comparison with the international experience, some countries in Africa had a striking absence of overwhelmed hospitals struggling to cope with surges of patients with severe COVID-19.

The media reported mass graves in New York, USA^4^ and Manaus, Brazil,^5^ but not in countries such as Kenya or Nigeria, despite modelled predictions of high numbers of deaths.^6^ Notable exceptions, where hospital surges were frequently seen, included north African countries such as Egypt, Morocco, and Tunisia, and South Africa.^7,8^ The contrast between some African countries and the severe hospital surges in Europe, the Americas, and Asia was regarded as “puzzling”^9^ and a “paradox”.^10^ Explanations put forward included the early and stringent public health interventions, altered transmission due to climate or ventilation, pre-pandemic population immunity, the young population, and poor surveillance systems having led to erroneous data. In this Review, we reflect on these possibilities with particular reference to observations made on the ground in Kenya, where the authors worked throughout the COVID-19 pandemic. We consider published data on initial transmission (ie, serological surveys), hospital admission data, demographic surveillance for mortality, and routine testing data, and contextualise these observations with work done outside of Africa.

### How rapid and how widespread was SARS-CoV-2 transmission in Africa?

One potential explanation is that the transmission rate of SARS-CoV-2 was low in parts of Africa because of either environmental factors or human factors. Specific explanations might include climate and seasonal effects on viral persistence in the environment,^11^ early or stringent public health interventions, improved air circulation in rural environments, and social policy.^12^ Substantial reductions in the rates of person-to-person transmission of SARS-CoV-2 would support such explanations.

In Kenya, figures from PCR testing in routine surveillance might have appeared to indicate low person-to-person transmission; 3 months after the first Kenyan case of COVID-19, only 20 000 cases had been identified (ie, affecting <0·1% of the population).^13^ However, a contemporaneous nationwide serological survey of blood donors using a well validated assay^14,15^ indicated that exposure was more widespread, with anti-SARS-CoV-2 antibodies present in 4·3% of the population.^16^ In contrast, 9 months after the first cases were seen in the UK, anti-SARS-CoV-2 antibody prevalence ranged from 3% to 10% depending on geography.^17^ Both the Kenyan and the UK figures would have been affected by lockdowns and many other factors; nevertheless, the comparison indicates that the rates of viral spread in Kenya and the UK were similar. Additional serological studies in Kenya indicated that antibody prevalence reached 22% in Nairobi, the capital city, by 6 months after the first COVID-19 case was reported.^18^ Community-based surveys done a year later indicated antibody prevalences in diverse geographical settings of 24–50% of the Kenyan population;^19^ surveys in 2022 found antibody prevalences of 72–94% of the population, indicating nearly universal exposure in some geographical locations.^20^ Our data on rapid spread in Kenya is consistent with antibody surveys from Malawi,^21^ Ethiopia,^22^ and Nigeria.^23^

In Kenya, we developed transmission models that incorporated these serological surveys and PCR testing. We found that initial transmission in Kenya was focused on less privileged socioeconomic groups in urban centres, and that transmission subsequently spread to more privileged socioeconomic groups in urban centres and finally to rural areas later in the epidemic.^24^ These findings and similar findings from a preprint paper in South Africa^25^ probably indicate that the ability to adhere to social restrictions depended on socioeconomic privilege, which resulted in an early so-called double peak of cases in Kenya.^24^ Subsequent peaks in transmission occurred due to the introduction of new SARS-CoV-2 variants, which spread widely through the population.^26,27^

The rapid spread of COVID-19 through urban and rural environments in Kenya excludes climate, outdoor living, lack of population mixing, social interventions, or other factors acting on viral transmission as the primary explanation for the absence of hospital surges in Kenya.

A variation on this group of explanations is that smaller viral inoculums (eg, in outdoor, well-ventilated settings) might still lead to infections, albeit with reduced severity. The link between inoculum and severity is supported by animal models;^28^ however, conditions leading to systematically smaller inoculum sizes would necessarily imply reduced infectiousness, which seems inconsistent with the evidence of rapid spread of SARS-CoV-2 through both rural and urban Kenyan populations.^18,20^ Furthermore, at least 40% of the population in sub-Saharan Africa live in urban conditions.^29^

## What symptoms did SARS-CoV-2 cause in the community?

The most common form of COVID-19 reported worldwide is a self-limiting febrile illness with respiratory symptoms. High numbers of symptomatic cases were reported in South Africa and in north African countries through routine surveillance. In Kenya, we found that the prevalence of asymptomatic infection among participants having routine testing was as high as 97·4% between March 17, 2020, and June 30, 2021.^30^ Furthermore, 7149 (7·4%) of 97 124 COVID-19 tests from asymptomatic participants were positive compared with 588 (22·9%) of 2568 tests from participants with symptoms, which suggests a very high prevalence of asymptomatic infection. However, routine data might be biased by treatment-seeking behaviour (eg, if most symptomatic individuals avoid testing, but asymptomatic individuals are tested in high numbers).

Active surveillance for SARS-CoV-2 was done in a vaccine trial^31^ that included seven scheduled clinic visits for 400 participants; this trial identified 87 cases of SARS-CoV-2 infection, all of which were asymptomatic. Furthermore, in a longitudinal study of 119 households with index cases, 43 (36·1%) were found to have secondary cases. 38 individuals with secondary cases were followed up for two weeks, during which 8 (21·1%) reported symptoms in their daily symptom diaries.^32^ All but one of these symptoms were limited to a single day’s duration, and the prevalence of symptoms was similar among individuals with a positive PCR test (8 [21·1%] of 38 individuals) and those with a negative PCR test (14 [18·2%] of 77). Considering the routine data and these active cohort studies, we conclude that the majority of SARS-CoV-2 infections in Kenya were asymptomatic.

Data from Kenya are consistent with data from Nigeria, where 75% of infections in routine testing data were asymptomatic,^33^ and symptoms were found to be poorly predictive of SARS-CoV-2 infection in contact-tracing studies.^34^ Frequent asymptomatic infections were also noted in community surveys in Malawi,^35^ and in a community survey in Guinea-Bissau.^36^ The prevalence of asymptomatic infection was much lower outside Africa, where approximately half of all infections appeared to be asymptomatic.^37,38^ In contrast to the effect of age on the risk of severe disease, age has only a modest effect on the risk of non-severe symptomatic disease. For instance, in meta-analyses, 47% of younger adults (aged 19–59 years) and 32% of children (aged 0–18 years) have asymptomatic infection compared with 20% of adults older than 60 years,^37^ hence the younger average age in Kenya would not explain the more than 80% prevalence of asymptomatic infection in both routine testing data and longitudinal cohort studies.

The prevalence of asymptomatic infection was 45% in South Africa,^39^ 38% in Egypt,^40^ and 30% in Madagascar,^41^ which indicates that the high prevalence of asymptomatic infection seen in Nigeria, Malawi, and Kenya did not generalise to all countries in Africa.

Post-COVID-19 condition (also known as long COVID) causes substantial morbidity in high-income settings. Asymptomatic infection might give rise to long COVID; however, definitions of long COVID are contested and most reports have depended on identifying symptomatic infection.^42^ Studies on long COVID in Africa are scarce. Two recent systematic reviews identified very few studies outside north Africa and South Africa, leaving substantial uncertainty about the long-term social and economic consequences of infection for many African populations.^43,44^

## Were there hospital surges due to COVID-19 in Africa?

Overwhelming surges of COVID-19 admissions occurred around the world, including in the USA,^45^ Europe,^46^ and South Africa.^7^ Case series of severe COVID-19 were recorded in countries in the rest of Africa, including Kenya^47^ and Malawi,^48^ and isolated health-care worker deaths were reported,^49^ but without a detectable increase in total hospital admissions.^50^ A study including hospitals in the Democratic Republic of the Congo, Ghana, Kenya, Nigeria, South Africa, and Uganda indicated that most children or adolescents with severe COVID-19, among whom mortality was 8%, had substantial comorbidities.^51^ Systematic and prospective data covering all admissions from 13 hospitals in Kenya indicated that public hospitals had consistent patient numbers throughout the pandemic waves.^52^ Six of these hospitals were designated COVID-19 treatment centres, and all 13 were typical district-level health facilities, including the coast and central and western Kenya. We noted a modest increase in the diagnosis of severe acute respiratory illness coinciding with periods of increased COVID-19 transmission in the community, but found no corresponding increase in overall patient numbers or any increase in mortality among those admitted. Furthermore, no evidence was found that hospital surges were masked by compensatory displacement of other activity; despite initial disruption of routine community services such as vaccination and malaria prevention due to physical distancing policies, activity rapidly returned to normal, including so-called catch up campaigns.^53–55^ Given the public interest in the pandemic, the reality of collapsing health care is unlikely to have been completely hidden from the media,^56^ and we consider it unlikely that hidden hospital surges occurred across Africa.

## Was there a high death rate among those who did not access hospital care?

Access to health care is low in many parts of Africa, and complete estimates of mortality therefore require data from the community as well as hospital surveillance.^57^ The balance of community versus hospital disease burden might be further shifted to the community by fear associated with COVID-19 leading to hospital avoidance,^58^ and might explain the so-called COVID-19 paradox in Africa if the low hospital numbers are associated with substantial undetected disease in the community. The likely outcome of absent hospital care for severe COVID-19 would be high death rates; however, although mass graves were reported in New York, USA^4^ and Manaus, Brazil,^5^ no similar reports came from Africa. Nevertheless, given the scarce vital registration data, under-reporting of mortality in the community was considered a possible explanation.^59^

Models to predict mortality in the absence of vital registration were constructed using parameters estimated from outside Africa or from north African countries and South Africa. One model indicated more than a million deaths in sub-Saharan Africa^60^ and another indicated more than a million in east Africa alone.^61^ Furthermore, some direct observations were supportive of high death rates in Africa. A study in Zambia used post-mortem PCR testing in Lusaka Hospital and found a high prevalence of SARS-CoV-2 virus;^62^ however, no contemporaneous data on the community prevalence of SARS-CoV-2 in Lusaka could be used to calculate attributable mortality. For instance, 20% of all deaths in patients aged 10–19 years in Lusaka Hospital were associated with PCR positivity. Given the rarity of death due to COVID-19 in this age range, most of the positive results in this age range would be more likely to indicate coincidental infection rather than the proximate cause of death. We cannot know the extent to which this was true of older age groups.

Direct estimates of excess deaths were generally considered to not be possible using the few civil registration systems available.^63,64^ In place of complete civil registration, research centres have demographic surveillance systems (DSSs) for sentinel surveillance of births and deaths.^65,66^ DSS studies in The Gambia^67^ and Kilifi County, Kenya have been used to estimate excess deaths during the pandemic.^68^

In Kenya, among more than 300 000 residents of the Kilifi DSS aged 1 year or older, 2441 observed deaths and 2276 expected deaths were recorded, resulting in an excess mortality rate approximately twice the 5400 deaths identified from routine surveillance in official statistics.^13^ Generalising data from Kilifi to the rest of Kenya is vulnerable to geographical heterogeneity. Kilifi County appears to have had average social vulnerability and below-average epidemiological vulnerability to COVID-19 compared with the rest of Kenya.^69^ Parallel figures from The Gambia showed a consistent pattern: in DSS studies covering a population of more than 250 000 residents in Basse, Farafenni, and Keneba, observed deaths ranged from 1438 to 1606 per year before the COVID-19 pandemic and were 1634 during 2020.^67^ In Madagascar, on the basis of death surveillance in the five districts of Antananarivo, 1179 excess deaths were reported.^70^

As seen in the table, in Kilifi County, The Gambia, Madagascar, and South Africa, the excess mortality calculated from community surveillance suggests more deaths than those identified by health service surveillance of COVID-19-related deaths. In low-income settings, routine health-care surveillance underestimates COVID-19 mortality due to missing data. Furthermore, excess mortality includes the sum of deaths caused directly or indirectly by COVID-19, deaths related to associated health-care or economic effects, and deaths from other unrelated causes. Combinations of these latter factors have been proposed to explain the patterns of excess mortality in high-income settings. However, in Kenya, South Africa, and Madagascar, where substantial excess mortality was identified, the timing of deaths aligned with waves of COVID-19, implicating underestimated mortality in health-care surveillance as the primary explanation for the gap.

Overall, in The Gambia and in Kilifi County, Kenya, the excess mortality calculated from community surveys was substantially lower than the predictions from international models. In Madagascar, the excess mortality from community surveys was in the low range of predictions from models. In South Africa, the community surveys and model predictions were aligned (table).

## Could the lower average age in Africa explain the mortality difference?

The average ages of the populations of the USA, South Africa, and Kenya are currently estimated as 38·9 years, 26·9 years, and 19·6 years, respectively.^73^ The infection fatality rate due to COVID-19 varies markedly by age. International data indicated that the infection fatality rate was 0·01% for ages 25–29 years, 0·1% for ages 40–45 years, and 1% for ages 65–70 years.^74^ The theoretical mortality from a single COVID-19 exposure to the Kenyan population can be calculated from the multiplication of infection fatality rates by the Kenyan population age structure, which provides a figure of approximately 50 000 deaths. On one hand, this figure might indicate reduced susceptibility a priori, as 50 000 is, for instance, substantially lower than the 200 000 deaths recorded in the UK despite access to vaccination and other mitigations; the overall population sizes of Kenya and the UK are similar (ie, 60·6 million in Kenya *vs* 69·5 million in the UK), but 5% of Kenyans are older than 60 years, compared with 18% in the UK.^13^ On the other hand, 50 000 is substantially higher than the 5400 deaths implied by generalising the DSS surveillance.

A calculation based on a single exposure of the population might be mitigated by data showing that the first SARS-CoV-2 infection accounted for the majority of severe disease^75,76^ and by data showing widespread serological evidence of exposure in Kenya.^24^ However, the infection fatality rates were derived from a population that made very substantial demands on its health-care systems. Specific health-care interventions reduce the mortality due to COVID-19^77,78^ and the more general effect of health care is evident through increased mortality when the hospital surge capacity is exceeded.^45,46^

The parallel calculation of multiplying infection hospitalisation rates by the age structure of the Kenyan population predicts a total of more than 1 million hospital admissions with severe COVID-19.^79^ Capacity in the Kenyan health-care system is substantially below these numbers^80^ and, as described previously, Kenya had no indication of substantial hospital surges.^52^ African countries might have less prevalent comorbidity than high-income settings, but some relevant comorbidities, such as tuberculosis, malnutrition, and HIV, are more prevalent in Africa.^81^ In Kenya, we noted a substantial prevalence of risk factors, including HIV (4·9%), diabetes (2·4%), hypertension (23·8%), and obesity (27·0%).^69^ Models that adjusted for comorbidity prevalence in Kenya still predicted nearly 1 million admissions to hospital with COVID-19 in Kenya.^82^

At the onset of the pandemic, it might have been argued that the age structure in Kenya and many other African countries would predictably lead to reduced mortality compared with European countries; however, it would have been unsafe to entirely base policy on this assumption without very substantial investment to increase surge capacity in the health system. Furthermore, the absence of the hospital surges and the even lower mortality observed in Kenya suggest that age structure is only a partial explanation of the COVID-19 paradox.

## Are there plausible biological explanations for a less marked host response?

The high prevalence of asymptomatic infection, the paucity of severe disease leading to hospital surges, and the low mortality could suggest a reduced host propensity to developing mild or severe disease at the centre of the COVID-19 paradox in Kenya. In contrast, asymptomatic SARS-CoV-2 infection was less prevalent in Madagascar and South Africa, where higher mortality was observed.^39,41,70,71^

Genetic factors are well established as risk factors for severe COVID-19,^83^ and HLA markers are associated with asymptomatic infection.^84^ However, in addition to the variation in outcomes across Africa, an increased risk of hospitalisation and death was seen among populations of African descent in the USA and in Europe in 2020.^85–87^ Therefore, genetic factors are unlikely to be a primary explanation for the epidemiology of COVID-19 seen in Kenya and elsewhere in Africa.

Vitamin D has been associated with less severe COVID-19 and might be considered a potential reason for lessened mortality in Africa. However, the protective efficacy of vitamin D status is modest,^88^ the direction of causality has been contested,^89^ and vitamin D status is not uniformly high in populations across Africa.^90^

The diagnostic antibody surveillance detailed previously found a very low prevalence of anti-SARS-CoV-2 antibodies before the COVID-19 pandemic.^15,16^ More detailed studies showed some evidence of cross-reactive neutralising antibodies before the pandemic in Kenya,^91^ but these cross-reactive antibodies were also found in European populations^92^ and were rare in both Kenya and Europe.

More data are needed on T-cell responses to SARS-CoV-2 in Africa, as there are very few studies on pre-pandemic responses, in contrast to studies in high-income countries where pre-pandemic T-cell responses were linked with protection.^93^ Pre-pandemic T-cell responses might have been acquired by previous exposure to an infectious agent circulating predominantly in Africa with cross-reactive T-cell epitopes, and studies in Uganda^94^ and a preprint paper published in Kenya^95^ have found high frequencies of pre-pandemic T cells reactive to SARS-CoV-2 peptides. Alternatively, previous exposures might affect susceptibility through bystander effects that reduce proinflammatory responses or promote regulatory responses.^96,97^ Furthermore, reduced inflammatory responses were seen among Senegalese patients with COVID-19 compared with European patients, including an absence of inflammasome and neutrophil activation.^98^ Other studies report cytokine profiles consistent with these findings in Malawi,^99^ Ghana,^100^ and Kenya (in a preprint paper).^101^

The distribution of high COVID-19 mortality in north African countries and in South Africa and low mortality on the rest of the continent also requires explanation, and the geographical distribution of risk led some to suggest that *Plasmodium falciparum* malaria generated immunity against COVID-19.^102^ However, central Kenya has very low malaria transmission, and hospitals in central Kenya showed no evidence of higher COVID-19 caseloads than hospitals in the west of Kenya, which has high malaria transmission according to evidence from a preprint paper.^103^ Furthermore, malaria endemicity is higher in Madagascar where COVID-19 outcomes appear to have been worse than in Kenya.

Besides malaria, other known and unknown infectious exposures could have distinct biogeography^104^ and might share epitopes with SARS-CoV-2 or have bystander effects on inflammatory responses.^105,106^ Other potential factors for more severe outcomes on comparison of north African countries and South Africa with Kenya or The Gambia include older and more comorbid populations.^107,108^

We hypothesise that a contributory explanation for the epidemiology seen in countries with less severe COVID-19 outcomes could be pre-pandemic multiple infectious exposures that lead to cross-reactive T-cell responses (but not cross-reactive antibodies), the outcome of which is less inflammatory cytokine responses on exposure to SARS-CoV-2. These less inflammatory responses could result in a high frequency of asymptomatic infection but nevertheless allow transmission to proceed. In South Africa and Madagascar, higher mortality and morbidity were reported and asymptomatic infection was less frequent than in other African countries such as Kenya or The Gambia. We therefore speculate that this variance in mortality and morbidity might in part be explained by variance in inflammatory cytokine responses across Africa. Furthermore, although we have identified data from some countries that indicate epidemiological outcomes that diverged from expectations, the data are patchy and insufficient to generalise across Africa.

## How did the COVID-19 pandemic compare with the 1918 influenza pandemic?

Whatever the explanation was for resilience to COVID-19, it does not appear to have occurred for the 1918 influenza pandemic. Records show devastating mortality in 1918 across Africa,^109^ including in Nigeria^110^ and Kenya,^111^ and the records of high mortality in 1918 in Kilifi sharply contrast with our data from the same county during the COVID-19 pandemic.^112^ Another contrast between the 1918 and 2020 pandemics was the inverse relationship with age (ie, higher mortality in young adults compared with older adults in 1918^113^ *vs* lower mortality in young adults in 2020). Hence, it seems prudent that policy makers faced with scarce data to guide them as COVID-19 spread to Africa took early firm action. Restrictions initially interrupted other public health activities,^114,115^ including malaria control;^116^ however, evidence suggested that public health activities rapidly rapidly recovered as restrictions were lifted,^53,54^ which shows the value of responding to local data.^117,118^

## Conclusion

The data included in this Review suggest a reduced susceptibility to severe illness and mortality associated with SARS-CoV-2 infection in Kenya, and probably in many other African countries, compared with Europe, North America, and other high-income countries. A low average age might be an important but incomplete explanation. The prevalence of asymptomatic infection and data linking asymptomatic infection to HLA types and cross-reactive T-cell responses could suggest pre-pandemic immunity due to other infectious agents leading to an attenuated inflammatory response on infection with SARS-CoV-2.

Health security in Africa remains fragile for future pandemics,^3^ for which rapid acquisition of data on the ground should be prioritised over externally led models and analysis generalising from external sources. Data in low-income settings are patchy and require prioritisation of the most informative and cost-effective surveillance modalities. Effective vital registration systems, demographic surveillance at sufficient scale to monitor mortality, and sentinel hospital surveillance provide essential data to guide pandemic responses. A focus on local data will facilitate better-informed models, cost-effective policy, and public health policy that evolves with the reality on the ground rather than external perceptions.

## Figures and Tables

**Table:** Observed and estimated COVID-19 deaths and mortality rates per location, 2020–21

	Identified by direct observation			Modelled estimates			
	DSS (deaths per 100 000 person-years)^67,68,70,71^	Predicted national excess deaths^67,68,70,71^	Routine surveillance deaths^13^	WHO AFRO^72^	WHO^60^	The Economist^6^	IHME^61^
Kilifi County, Kenya	31·0*	13 700*	5400	17 000	28 000	117 000	171 000
The Gambia	11·1	308	343	1450	1578	3442	6340
Madagascar	38·51 (2020) and 64·91 (2021)	30 000	1426	9822	46 642	46 737	65 100
South Africa	NA	~300 000	91 564	92 118	247 000	273 110	302 000

South African data to predict national excess mortality depend on vital registration rather than DSS results. AFRO=Regional Office for Africa. DSS=demographic surveillance system. IHME=Institute for Health Metrics and Evaluation. NA=not applicable.

* Figure relates to people aged 1 year or older.

## Key messages

- COVID-19 in Africa did not result in the morbidity and mortality across the entire continent that was expected. We review the available evidence to assess whether reduced viral transmission, public health interventions, poor surveillance, or biological explanations are plausible explanations.
- High mortality and morbidity were well documented in some African countries such as South Africa and north African countries. Data are scarce across the rest of Africa, but community surveys in Kenya and in The Gambia indicate low mortality, and data from Madagascar indicate intermediate mortality.
- Evidence of widespread viral transmission was consistent with observations outside Africa.
- Population age structure is an important but incomplete explanation of the reduced mortality.
- The high prevalence of asymptomatic infection and low mortality, together with evidence of reduced inflammatory responses, could indicate that some populations in Africa have reduced susceptibility to symptomatic COVID-19.
- When responding to global pandemics, local data are essential to develop public health policies that align with the reality on the ground rather than external perceptions.

## Search strategy and selection criteria

We searched PubMed and Google Scholar from database inception to Nov 11, 2024, using the terms “Transmission”, “Serology”, “Testing”, “Asymptomatic” “Excess Mortality” “DSS” or “Surveillance”, combined with the following Boolean search terms: AND (“COVID” OR “SARS-CoV-2”) AND (“Africa”). For key commentaries, we examined referenced articles and citing articles using Google Scholar. We included press articles to illustrate selected points without systematic searches. Only English language articles were included. Inclusion criteria were not systematic and articles were included based on relevance to the narrative review.
